# SARS-CoV-2 Isolates Show Impaired Replication in Human Immune Cells but Differential Ability to Replicate and Induce Innate Immunity in Lung Epithelial Cells

**DOI:** 10.1128/spectrum.00774-21

**Published:** 2021-08-11

**Authors:** Miao Jiang, Pekka Kolehmainen, Laura Kakkola, Sari Maljanen, Krister Melén, Teemu Smura, Ilkka Julkunen, Pamela Österlund

**Affiliations:** a Expert Microbiology Unit, Department of Health Security, Finnish Institute for Health and Welfare, Helsinki, Finland; b Infection and Immunity, Institute of Biomedicine, University of Turku, Turku, Finland; c Medicum, Department of Virology, University of Helsinki, Helsinki, Finland; d Clinical Microbiology, Turku University Hospital, Turku, Finland; Karolinska Institutet

**Keywords:** SARS-CoV-2, COVID-19, viral replication, human macrophage, human DC, Calu-3 cell, Vero E6 cell, TPCK-treated trypsin, interferon (IFN) response

## Abstract

The primary target organ of coronavirus disease 2019 (COVID-19) infection is the respiratory tract. Currently, there is limited information on the ability of severe acute respiratory syndrome coronavirus 2 (SARS-CoV-2) to infect and regulate innate immunity in human immune cells and lung epithelial cells. Here, we compared the ability of four Finnish isolates of SARS-CoV-2 from COVID-19 patients to replicate and induce interferons (IFNs) and other cytokines in different human cells. All isolates failed to replicate in dendritic cells, macrophages, monocytes, and lymphocytes, and no induction of cytokine gene expression was seen. However, most of the isolates replicated in Calu-3 cells, and they readily induced type I and type III IFN gene expression. The hCoV-19/Finland/FIN-25/2020 isolate, originating from a traveler from Milan in March 2020, showed better ability to replicate and induce IFN and inflammatory responses in Calu-3 cells than other isolates of SARS-CoV-2. Our data increase the knowledge on the pathogenesis and antiviral mechanisms of SARS-CoV-2 infection in human cell systems.

**IMPORTANCE** With the rapid spread of the coronavirus disease 2019 (COVID-19) pandemic, information on the replication of severe acute respiratory syndrome coronavirus 2 (SARS-CoV-2) and regulation of innate immunity in human immune cells and lung epithelial cells is needed. In the present study, we show that SARS-CoV-2 failed to productively infect human immune cells, but different isolates of SARS-CoV-2 showed differential ability to replicate and regulate innate interferon responses in human lung epithelial Calu-3 cells. These findings will open up the way for further studies on the mechanisms of pathogenesis of SARS-CoV-2 in human cells.

## INTRODUCTION

In the beginning of December 2019, several patients in Wuhan, China, were hospitalized with symptoms of pneumonia as the start of an outbreak of coronavirus disease 19 (COVID-19), named by the WHO. The previously unknown causative virus was later named severe acute respiratory syndrome coronavirus 2 (SARS-CoV-2) due to its high sequence similarity with the 2002 SARS-CoV. Within 3 months, COVID-19 spread all around the world, and as of July 2021, over 185 million cases and 4 million deaths have been confirmed worldwide, and the numbers of cases and deaths are still rising.

Coronaviruses (CoVs; subfamily *Orthocoronavirinae*) are the largest group of RNA viruses that belong to the family *Coronaviridae* (1). They are enveloped, pleomorphic positive-sense single-stranded RNA viruses with a genome size ranging from 26 to 32 kb (2). CoVs were first found in infectious bronchitis virus-infected chickens in the 1930s (3). CoVs can be transmitted among different animal species by spillover events (2). To date, hundreds of coronaviruses have been characterized, with most of them circulating among animals, such as mice (4), pigs (5), cats (6), camels (7), ferrets (8), bats (9), and other animal species. Some of the CoVs are zoonotic, and thus they may be transferred from other vertebrate species to humans and cause disease (10, 11). Currently, there are seven species of CoVs that can cause upper and lower respiratory tract infections in humans. HCoV-229E (229E) and HCoV-NL63 (NL63) belong to the *Alphacoronavirus* genus, while HCoV-OC43 (OC43) and HCoV-HKU1 (HKU1) belong to the *Betacoronavirus* genus; these four strains are common coronaviruses circulating in humans during winter and spring-summer seasons and cause mainly mild symptoms (12). However, the remaining three betacoronaviruses, SARS-CoV (13, 14), Middle East respiratory syndrome-related coronavirus (MERS-CoV) (13, 14), and SARS-CoV-2 (15) may cause severe symptoms, such as pneumonia and acute respiratory distress syndrome (ARDS), which may lead to death. Bats have been reported to serve as the likely natural reservoir for these three CoVs (9, 16); however, SARS-CoV and MERS-CoV were transmitted to humans via the intermediate hosts civet cats and dromedary camels, respectively (17). The possible intermediate host of SARS-CoV-2 has not yet been confirmed. However, pangolins have been speculated to be able to function as a potential intermediate host (18, 19). The outbreaks of SARS in China (2002) and MERS in Saudi Arabia (2012) have led to sporadic cases and limited epidemic clusters with a mortality rate of 9.6% and 34%, respectively (20). SARS-CoV-2 appears to be clearly more contagious than SARS-CoV and MERS-CoV, and it has efficiently spread throughout the world (21).

As respiratory pathogens, SARS-CoV and SARS-CoV-2 enter the host through the respiratory tract. The airway epithelium, including lung epithelial cells, constitutes the first line of host defense against invading pathogens. Underneath the alveolar epithelium, macrophages (MΦs) and dendritic cells (DCs) are abundantly present especially in virus-infected lungs, and they act as the key cell types regulating innate immunity. Innate immunity plays a crucial role at early stages of viral infection, regulating its spread. In addition to interacting with pathogens and mediating acute inflammatory responses, MΦs and DCs also bridge innate and adaptive immune responses during infections (22). Although balanced early innate immune responses are beneficial in facilitating pathogen clearance, destabilized and excessive cytokine production by immune cells can lead to exacerbated inflammatory responses known as the “cytokine storm” (23). This has been reported to result in severe tissue damage in highly pathogenic avian influenza A (H5N1) virus (24), SARS-CoV (25), and SARS-CoV-2 (26) infections. Therefore, lung epithelial cells, MΦs, and DCs are the pivotal cell types responding to invading pathogens and regulating the outcome of the infection.

Still, the pathogenic mechanisms of SARS-CoV-2 have not fully been revealed; in particular, there is limited information on the potential differences of SARS-CoV-2 strains in their ability to replicate and induce innate immunity in human cells. It has previously been shown that SARS-CoV and MERS-CoV infections in human MΦs and DCs are abortive, yet some innate immune response is induced (27, 28). In the present study, we have isolated SARS-CoV-2 strains from nasopharyngeal samples of COVID-19 patients in Finland in Spring 2020 and characterized the infection of four isolates of SARS-CoV-2 in primary human DCs, MΦs, monocytes, and lymphocytes as well as in the human lung epithelial cell line Calu-3. In addition, we have investigated the ability of different isolates of SARS-CoV-2 to induce innate immune responses.

## RESULTS

### Isolation, culturing, and genomic comparison of four strains of SARS-CoV-2 from patient samples.

**FIG 1:**
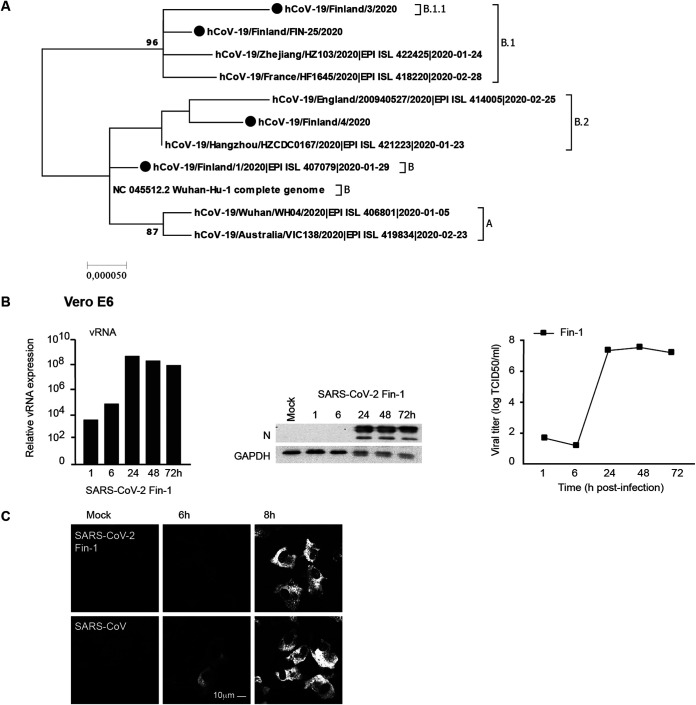
Phylogenetic analysis of four SARS-CoV-2 isolates and replication of SARS-CoV-2 in Vero E6 cells. (A) Phylogenetic analysis of the four first Finnish SARS-CoV-2 isolate complete sequences with relevant reference sequences (*n* = 11 sequences). The reference sequences include the reference strain, Wuhan-Hu-1, as well as six sequences from isolates representing major lineages A, B, B.1, B.1.1, and B.2 of SARS-CoV-2 sequences. (B) The first Finnish SARS-CoV-2 isolate, Fin-1, was used at an MOI of 0.1 TCID_50_/cell to analyze its infectivity in Vero E6 cells. Total cellular RNA, protein, and cell culture supernatants were collected, and viral RNA (vRNA) and viral N protein expression levels as well as the viral titers during the 3-day infection were determined. (C) The comparison to the SARS-CoV infection by viral N protein expression was analyzed at 6-h and 8-h time points with the SARS-CoV-2 Fin-1 strain in Vero E6 cells by immunofluorescence staining with cross-reactive anti-N (SARS-CoV) rabbit antisera; scale bar, 10 μm. Results are representative of two independent experiments.

The first COVID-19 case in Finland was confirmed on 29 January 2020 when a 32-year-old female Chinese tourist traveled from Wuhan to Lapland (29). The second COVID-19 case in Finland was confirmed on 26 February 2020 when a Finnish woman returned from Milan, Italy. Later on, COVID-19 started to spread rapidly in Finland from early March 2020. The first and second Finnish SARS-CoV-2 strains were isolated from patient nasopharyngeal aspirate samples in Vero E6 cells, and the viruses were named as hCoV-19/Finland/1/2020 (Fin-1) (29) and hCoV-19/Finland/FIN-25/2020 (Fin-25) (30), respectively. In March, just before the Finnish boarders were closed, two additional strains of SARS-CoV-2, hCoV-19/Finland/3/2020 (Fin-3) and hCoV-19/Finland/4/2020 (Fin-4), were isolated and propagated in the same way in Vero E6 cells. We sequenced the genomes of these four strains of SARS-CoV-2 both from the original swab samples and from cell-cultured viruses and compared the nucleotide and amino acid residue changes with the genome of the reference strain Wuhan-Hu-1 of SARS-CoV-2 (Table 1). The genome sequences of the strains of SARS-CoV-2 have been uploaded to the global initiative on sharing avian influenza data (GISAID) (EPI_ISL_407079, EPI_ISL_412971, EPI_ISL_2365908, and EPI_ISL_2365909). Fin-1 and Fin-25 strains differ from each other in three amino acid residues, one of which is situated in the RNA-dependent RNA polymerase (RdRp) and two of which are in the spike (S) protein (Table 1). The sequence of Fin-1 is nearly identical (1-nucleotide [nt] difference) with the Wuhan-Hu-1 reference strain, which belongs to B clade, whereas Fin-25, together with Fin-3, cluster together with sequences belonging to the B.1. and B.1.1 clades, respectively (Fig. 1A). The sequence of the Fin-4 strain belongs to the B.2 clade (Fig. 1A). Propagation of the viruses in Vero E6 cells lead to a 15-nt deletion (deletion of nt 23,583 to 23,597) or a single nt (C23606T, R682W) change in all of the virus isolates. However, the proportion of the virus progeny population having these changes varied between the isolates (Table 2). Both of these changes are close to the furin-like cleavage site in the S protein sequence.

**TABLE 1:** Nucleotide and amino acid residue changes of the Finnish isolates in comparison to the reference SARS-CoV-2 Wuhan-Hu-1 sequence

Isolate	Nt position^*a*^	Region^*b*^	Nt change	AA change	Lineages sharing the change
hCoV-19/Finland/1/2020	21,707	S	C → T	H49Y	
					
hCoV-19/Fin-25/2020	241	5′-UTR	C → T		B.1
	3,037	Nsp3^*c*^	C → T		B.1
	14,408	Nsp12	C → T	P314L	B.1
	23,403	S	A → G	D614G	B.1
	26,187	Orf3a	T → C		
					
hCoV-19/Finland/3/2020	241	5′-UTR	C → T		B.1
	3,037	Nsp3	C → T		B.1
	9,347	Nsp4	A → G	T3028A	
	14,408	Nsp12	C → T	P314L	B.1
	23,403	S	A → G	D614G	B.1
	28,881	N	G → A	R203K	B.1.1
	28,882	N	G → A		B.1.1
	28,883	N	G → C	G204R	B.1.1
					
hCoV-19/Finland/4/2020	11,083	Nsp6	G → T	L3606F	B.2
	14,805	Nsp12	C → T		
	18,086	Nsp14	C → T	T1540I	
	26,144	Orf3a	G → T	G251V	
	28,842	N	G → T	I190S	

a Nucleotide (nt) position is numbered according to the Wuhan-Hu-1 reference sequence.

b 5′-UTR, 5′-untranslated region; AA, amino acid; N, nucleoprotein; Nsp12, RNA-dependent RNA polymerase; S, spike protein; ORF3a, 3a open reading frame; Nsp14, 3′-5′ exonuclease.

c Nsp3-Nsp4-Nsp6 form a complex, which is involved in viral replication.

**TABLE 2:** Introduction of furin cleavage site affecting changes in the virus population following virus propagation in Vero E6 cells

		Percentage of the sequencing reads having this change		
Isolate	Sample	23,583–23,597 deletion (S gene)	23,606 C → T (aa R682W) (S gene)	Percentage of wild-type population^*a*^
hCoV-19/Finland/1/2020	Fin-1 swab sample	0%	0%	100%
	Fin-1 VE6 p3	78%	3%	19–22%
				
hCoV-19/Fin-25/2020	Fin-25 swab sample	0%	0%	100%
	Fin-25 VE6 p3	38%	32%	30–62%
				
hCoV-19/Finland/3/2020	Fin-3 swab sample	0%	0%	100%
	Fin-3 VE6 p2	33%	14%	53–67%
				
hCoV-19/Finland/4/2020	Fin-4 swab sample	0%	0%	100%
	Fin-4 VE6 p2	7%	42%	51–58%

a The percentages vary since the sequence analysis does not specify whether either one or both mutations reside in the same virus particle.

To identify SARS-CoV-2 cell tropism, we evaluated the ability of the virus isolates to infect and replicate in Vero E6 cells and in several human cell types. Vero E6 cells were first challenged with the SARS-CoV-2 Fin-1 strain (Vero E6, passage 3) at a multiplicity of infection (MOI) of 0.1. The results of quantitative reverse transcription PCR (qRT-PCR) with SARS E gene-specific probes showed that the expression of viral RNA was remarkably elevated already at 24 h postinfection (p.i.), remaining at high levels until 72 h p.i. (Fig. 1B). Western blotting analysis with cross-reactive anti-N protein (SARS-CoV) rabbit antisera (Fig. 1B) showed strong expression of viral N protein starting at 24 h p.i. To analyze the productivity of the infection, cell culture supernatants were collected, and viral titers at different time points p.i. were determined with an endpoint dilution assay (Fig. 1B). Consistent with the results from qRT-PCR and Western blotting, SARS-CoV-2-infected Vero E6 cells produced high levels of infectious viruses after 24 h p.i., with virus titers reaching levels of 10^7^ 50% tissue culture infective dose (TCID_50_)/ml (Fig. 1B). Immunofluorescence assays showed weak SARS-CoV-2 N protein expression in cells at 6 h p.i., while viral N protein expression in SARS-CoV-infected cells was more clearly detectable (Fig. 1C). N protein expression was observed in both SARS-CoV-2- and SARS-CoV-infected cells at 8 h p.i., suggesting practically similar replication kinetics of these viruses in Vero E6 cells (Fig. 1C).

### Replication of SARS-CoV-2 Fin-1 and Fin-25 in human primary immune cells.

**FIG 2:**
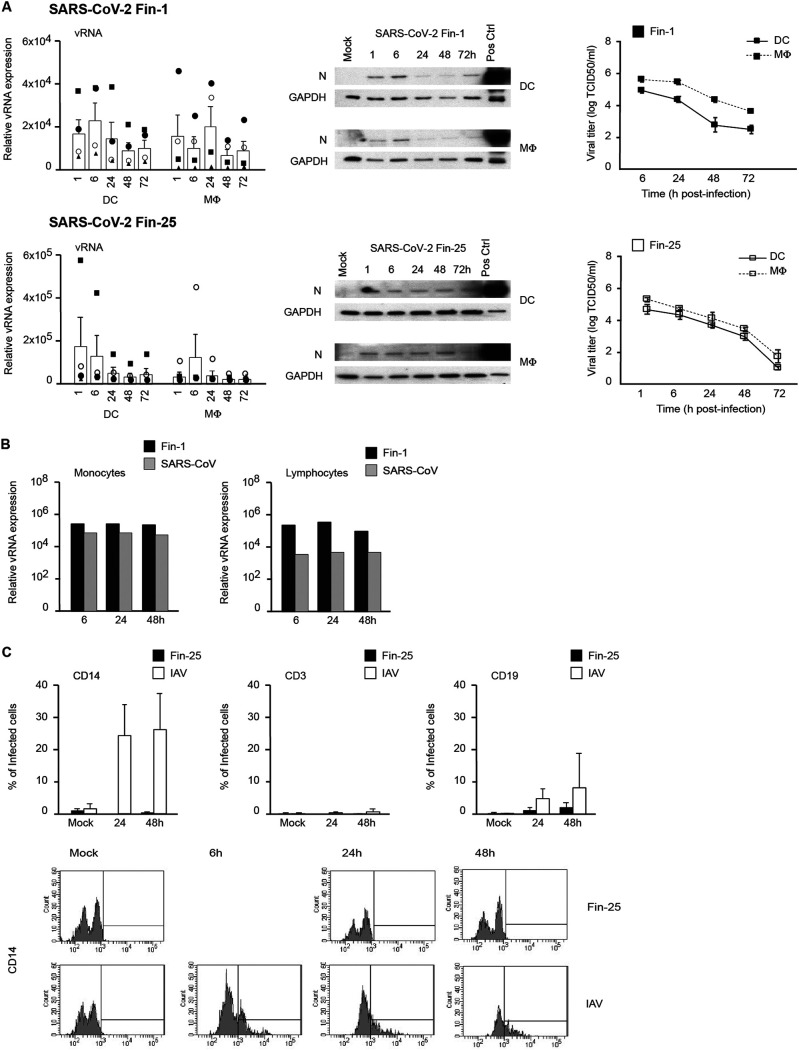
Susceptibility of human primary immune cells to SARS-CoV-2 infection. (A) Human monocyte-derived DCs and MΦs from four different blood donors were separately infected with SARS-CoV-2 Fin-1 and Fin-25 strains at an MOI of 1 TCID_50_/cell, and samples were collected at different times after infection as indicated in the figure. From total cellular RNA samples, viral RNA expression was analyzed using a SARS E gene-specific qRT-PCR assay. Individual donors are shown with different symbols, and the results are shown as relative copy numbers over the mock sample. Data are presented as the means ± SEM of cells generated from four independent blood donors. SARS-CoV-2-infected cells from the same four donors were collected and pooled for viral protein expression analysis and immunoblotted using anti-N (SARS-CoV) and anti-GAPDH antibodies. Fin-1-infected Vero E6 cell extract was used as a positive control (Pos Ctrl). From the same cell cultures, the supernatant samples were harvested for virus titrations in Vero E6 cells, and titers are shown as log TCID_50_/ml representing the means ± SEM from DC or MΦ cultures from four different blood donors. (B) Human monocytes and lymphocytes from four different donors were infected with SARS-CoV-2 Fin-1 and SARS-CoV (MOI of 1) for 6, 24, and 48 h, and cellular RNA samples from different donors were pooled and analyzed for the expression of viral RNA. The data are shown as a relative copy number over the mock sample. (C) Human PBMCs from four different blood donors were separately infected with the SARS-CoV-2 Fin-25 strain and IAV at an MOI of 1, and samples were collected at different times after infection as indicated in the figure. SARS-CoV-2- or IAV-infected cells were harvested, fixed separately, and double stained with indicated antibodies for specific cell surface markers and viral antigens. The samples were analyzed with a FACSCanto II (BD) device using FACSDiva software. Data are presented as the means ± SEM of cells generated from four independent blood donors. Representative histogram patterns from flow cytometric analysis of SARS-CoV-2 Fin-25 and IAV virus-infected CD14-positive cells from one donor is shown. A 6-h time point was included to the IAV-infected samples due to the faster infection kinetics.

Previous studies have shown that human monocyte-derived MΦs and DCs are nonpermissive to the replication of SARS-CoV and MERS-CoV (27, 28). As the ability of SARS-CoV-2 to spread among humans seems to be much higher than that of SARS-CoV or MERS-CoV, we addressed the question whether SARS-CoV-2 would also be able to infect and replicate in human primary immune cells. DCs and MΦs from four different blood donors were separately infected with Vero E6-cultured Fin-1 and Fin-25 strains of SARS-CoV-2 at an MOI of 1. qRT-PCR data on cells of individual donors showed that both Fin-1 and Fin-25 strains failed to replicate in DCs and MΦs, as the expression of viral RNA failed to increase and instead started to decrease 24 h after infection (Fig. 2A). The expression of viral N protein (input virus) was detected at 1 h and some later time points p.i. in Fin-1 and Fin-25 virus-infected cells, but viral protein expression failed to increase during the 72-h follow-up (Fig. 2A). Endpoint dilution assay carried out with the supernatants of Fin-1- or Fin-25-infected cells confirmed this observation, showing a clear decrease of virus titers during the infection in both DCs and MΦs (Fig. 2A). Similar experiments were also performed in monocytes and lymphocytes when they were challenged by an infection with SARS-CoV-2 (Fin-1) or SARS-CoV. qRT-PCR showed that the expression of viral RNA remained at a similar input level during the 2-day infection (Fig. 2B), indicating that these cells are nonproductively infected by SARS-CoV or SARS-CoV-2. To further demonstrate that SARS-CoV-2 is unable to infect subpopulations of human peripheral blood mononuclear cells (PBMCs) investigated above, fluorescence-activated cell sorting (FACS) analysis was performed in SARS-CoV-2 (Fin-25)-infected human PBMCs followed by staining with antibodies for specific cell surface markers (CD14 for monocytes, CD3 for T cells, and CD19 for B cells) and viral antigen (N for SARS-2 virus) (Fig. 2C). Influenza A virus (IAV) H3N2 strain was used as a positive control and was stained with IAV-specific rabbit antisera (Fig. 2C). The data indicated that IAV was able to infect human monocyte subpopulations and B cell subpopulations at some level, while SARS-CoV-2 could not infect any subpopulations of human PBMCs. The results of FACS analysis are consistent with those of qRT-PCR, Western blotting, and endpoint dilution assays, indicating that SARS-CoV-2 does not replicate in human macrophages, DCs, monocytes, or T or B cells.

### Replication of SARS-CoV-2 strains in Calu-3 cells.

**FIG 3:**
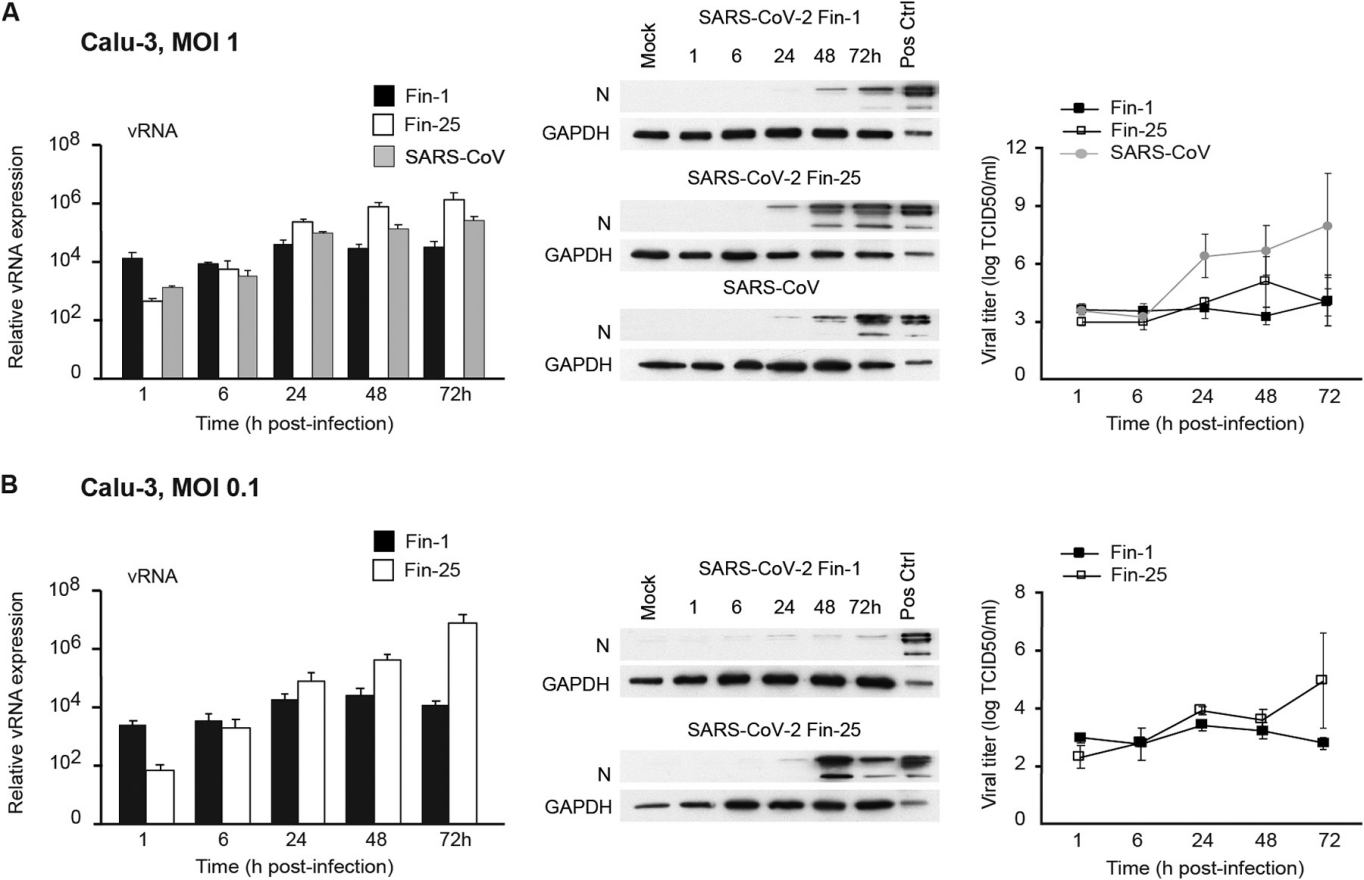
Comparison of SARS-CoV-2 isolates and SARS-CoV replication in Calu-3 cells. (A) Calu-3 cells were infected with SARS-CoV-2 Fin-1 or Fin-25 strains or SARS-CoV (MOI of 1). Virus replication was assessed at different time points after infection as viral RNA expression using SARS E gene-specific probes and as viral protein expression visualized by immunoblotting analysis using anti-N protein (SARS-CoV) antisera as well as viral titers from the supernatant samples. SARS-CoV-2-infected Vero E6 cell extract was used as a positive control in immunoblots where a representative experiment out of two is shown. For RNA and titration data, the results from three experiments are shown as mean values ± SEM. (B) Calu-3 cells were challenged with SARS-CoV-2 Fin-1 or Fin-25 at a low MOI of 0.1 TCID_50_/cell for different times. Virus replication was analyzed on the RNA and viral protein levels as well as by titrating the progeny viruses. The experiment was repeated three times, and the results are shown as the means ± SEM. One representative immunoblot is shown.

Calu-3 cells were further challenged with Vero E6-cultured Fin-1 and Fin-25 strains of SARS-CoV-2 and the 2003 SARS-CoV at an MOI of 1 TCID_50_/cell. The expression of viral RNA in Fin-25 virus-infected cells was strongly elevated already at 24 h p.i. The Fin-25 strain appeared to replicate slightly better than SARS-CoV and much better than the Fin-1 strain (Fig. 3A), suggesting different replication kinetics of Fin-1 and Fin-25 strains in Calu-3 cells. Western blotting analysis indicated that viral N protein expression appeared to occur earlier in SARS-CoV-2 Fin-25 strain-infected cells than in Fin-1 virus-infected cells (Fig. 3A). However, the level of produced infectious Fin-25 virus was similar to that of Fin-1 (Fig. 3A), suggesting that both strains of SARS-CoV-2 could not replicate productively in Calu-3 cells. Interestingly, even if 2003 SARS-CoV replicated at a slightly lower level than Fin-25 virus (RNA and N protein expression), the production of infectious virus was dramatically higher in SARS-CoV infection than in Fin-1 or Fin-25 virus infection (Fig. 3A). Similar results were found when Calu-3 cells were challenged with Fin-1 and Fin-25 strains of SARS-CoV-2 at a low MOI value of 0.1 TCID_50_/cell (Fig. 3B). This indicates that in Calu-3 cells, the Fin-1 strain can only induce weak expression of viral RNA and N protein but no production of progeny viruses, whereas the Fin-25 strain can induce stronger viral RNA and protein expression but still lacks a clear propagation of viral particles.

### Trypsin treatment enhanced the replication of SARS-CoV-2 strains in Calu-3 cells but not in human monocyte-derived DCs and MΦs.

**FIG 4:**
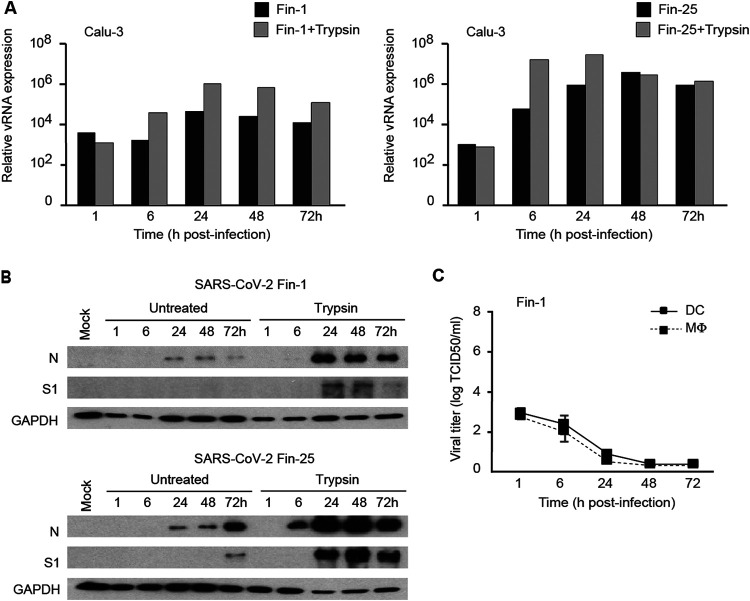
TPCK-treated trypsin treatment enhances SARS-CoV-2 infectivity in Calu-3 cells but not in DCs or MΦs. (A) Calu-3 cells were infected with SARS-CoV-2 Fin-1 and Fin-25 pretreated with TPCK-treated trypsin (35 μg/ml) or untreated viruses for 3 days. Viral RNA expression was analyzed from the total cellular RNA samples by viral E gene-specific qRT-PCR. One representative experiment out of two is shown. (B) The expression of SARS-CoV-2 viral proteins was analyzed in Calu-3 cells at different time points after infection with untreated Fin-1 or Fin-25 or viruses pretreated with TPCK-treated trypsin. Total cellular protein samples from virus-infected cells were prepared for immunoblotting against viral S1 and N proteins. GAPDH was used as a loading control. (C) Human primary DCs or MΦs from four different donors were separately infected with trypsin-pretreated SARS-CoV-2 Fin-1. Viral titers were analyzed from the cell culture supernatants at different time points after infection. Results are shown as log TCID_50_/ml and represent the means ± SEM from DCs or MΦs obtained from four different donors.

Recently, several studies have shown that the cleavage of S protein of SARS-CoV-2 by cellular proteases is essential for the fusion of viral and cellular membranes and facilitates the cell entry of the virus (31–33). Trypsin treated with tosylsulfonyl phenylalanyl chloromethyl ketone (TPCK) has been demonstrated to cleave S protein of several CoVs and increase the infection and replication of the virus in different cell types (34–38). To evaluate whether protease treatment of SARS-CoV-2 can enhance or facilitate virus replication in Calu-3 cells or in human primary cells, SARS-CoV-2 Fin-1 and Fin-25 stock viruses were pretreated with TPCK-treated trypsin (35 μg/ml). Calu-3 cells were infected with trypsin-treated or untreated viruses at an MOI of 1. The results of the qRT-PCR assay from SARS-CoV-2-infected Calu-3 cells showed that the pretreatment of viruses with TPCK-treated trypsin notably enhanced the replication of both Fin-1 and Fin-25 strains in these cells (Fig. 4A). Western blotting analysis showed that the pretreatment of viruses with TPCK-treated trypsin clearly enhanced the expression of S1 and N protein in both Fin-1- and Fin-25-infected Calu-3 cells (Fig. 4B). However, the pretreatment of viruses with TPCK-treated trypsin failed to change the abortive nature of the replication of SARS-CoV-2 in human DCs and MΦs, as the TCID_50_ values continued to decrease during the infection with the trypsin-pretreated SARS-CoV-2 Fin-1 strain (Fig. 4C).

### Activation of innate immune responses in SARS-CoV-2 Fin-1 and Fin-25 virus-infected Calu-3 cells.

**FIG 5:**
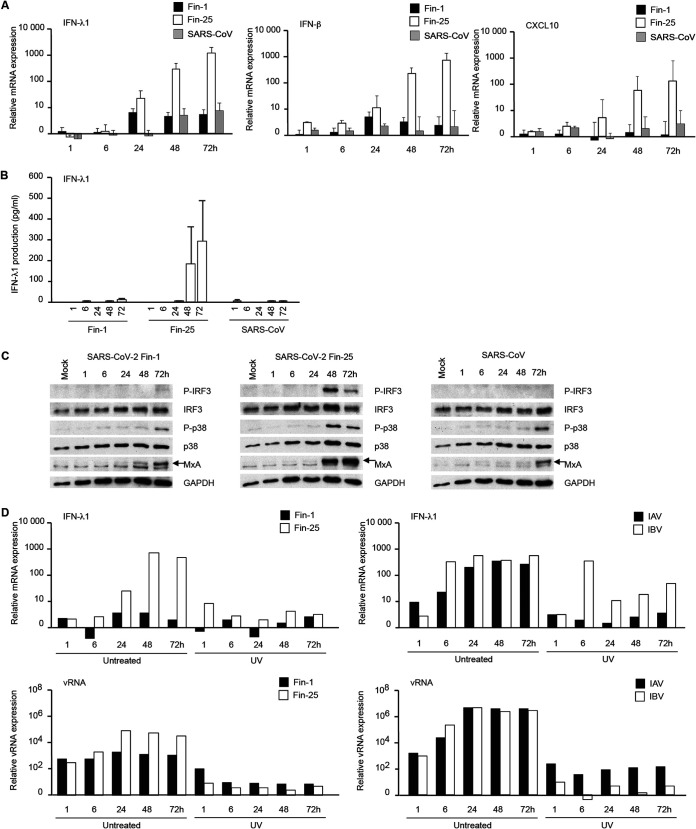
Comparison of SARS-CoV-2 Fin-1- and Fin-25- and SARS-CoV-induced antiviral cytokine responses in Calu-3 cells. (A) mRNA levels of antiviral cytokines IFN-λ1, IFN-β, and CXCL10 were analyzed at different time points after infecting Calu-3 cells with SARS-CoV-2 Fin-1 or Fin-25 or SARS-CoV (at an MOI of 1). The results are shown as a relative fold induction of each cytokine mRNA compared to levels observed in mock cells. The data represent mean values ± SEM of three independent experiments. (B) Secreted IFN-λ1 protein levels were analyzed by ELISA from Calu-3 cell culture supernatants collected at different times after infection. The supernatants from three independent experiments were analyzed separately, and the results (pg/ml) represent the mean values ± SEM. (C) The expression of antiviral signaling molecules was analyzed in Calu-3 cells at different time points after infection with Fin-1, Fin-25, or SARS-CoV viruses. Total cellular protein samples were prepared for immunoblotting against phospho-IRF3 (P-IRF3), IRF3, phospho-p38 (P-p38), p38, and antiviral MxA. GAPDH was used as a loading control. (D) Calu-3 cells were infected with live or UV-irradiated SARS-CoV-2 Fin-1 or Fin-25, IAV, or IBV (MOI 1), and samples were collected at different times after infection as indicated in the figure. From total cellular RNA samples, viral RNA expression was analyzed using SARS E gene-, IAV M1 gene-, or IBV NP gene-specific qRT-PCR assays. Expression of the antiviral cytokine IFN-λ1 gene was also analyzed by qRT-PCR at different time points after infection. The results are shown as a relative fold induction compared to levels observed in mock cells. One representative experiment out of three is shown.

Since the SARS-CoV-2 Fin-25 strain showed better viral RNA and protein expression than the Fin-1 strain and the 2003 SARS-CoV in Calu-3 cells, we addressed the question whether these viruses would differentially induce innate immune responses. We analyzed mRNA expression of cytokines interferon-α (IFN-α), IFN-β, IFN-λ1, chemokine (C-X-C motif) ligand (CXCL) 10, interleukin-1β (IL-1β), IL-6, IL-8, and tumor necrosis factor-α (TNF-α) by qRT-PCR from cellular RNAs isolated from SARS-CoV- and SARS-CoV-2 (Fin-1 and Fin-25 strains)-infected Calu-3 cells. Both Fin-1 and SARS-CoV failed to induce notable mRNA expression of IFN-λ1 (Fig. 5A). However, Fin-25 strains induced IFN-λ1 mRNA expression at 6 h p.i. onwards, and the expression continued to increase and showed an ∼1,000-fold increase over basal levels within 72 h (Fig. 5A). Moreover, the Fin-25 strain induced higher mRNA expression levels of IFN-β and CXCL10 than the SARS-CoV-2 Fin-1 strain or SARS-CoV (Fig. 5A). Still, both Fin-1 and Fin-25 viruses failed to induce notable expression of IFN-α, IL-1β, IL-6, IL-8, and TNF-α mRNA in Calu-3 cells (data not shown). We also quantitated IFN-λ1 production by enzyme-linked immunosorbent assay (ELISA) in the supernatants of SARS-CoV-2 (Fin-1 and Fin-25)- and SARS-CoV-infected Calu-3 cells. In accordance with the qRT-PCR results, clearly detectable amounts of IFN-λ1 protein were seen with Fin-25 infection at later time points (48 and 72 h p.i.), while IFN-λ1 production induced by Fin-1 or by SARS-CoV was not detectable (Fig. 5B). Western blotting analysis also showed that infection with the Fin-25 strain induced phosphorylation of interferon regulatory factor 3 (IRF3) and p38 in Calu-3 cells starting from 48 h p.i., while the infection with the Fin-1 virus induced very weak phosphorylation of p38 and IRF3 at 72 h p.i. (Fig. 5C). Infection with SARS-CoV failed to induce detectable phosphorylation of IRF3 in infected cells (Fig. 5C). However, all strains of CoVs induced the expression of type I and type III IFN-inducible myxovirus resistance protein 1 (MxA) protein at late stages of infection. Infection with Fin-25 virus, which was the best inducer of IFN-λ1, also induced the highest level of MxA protein expression (Fig. 5C). We also analyzed IFN-α, IFN-λ1, CXCL10, IL-1β, and IL-6 mRNA expression levels by qRT-PCR from total cellular RNA samples isolated from SARS-CoV- and SARS-CoV-2 (Fin-1 and Fin-25)-infected DCs and MΦs; however, no enhanced expression of these genes was observed (data not shown). Ultraviolet (UV)-irradiated Fin-1 and Fin-25 failed to replicate in Calu-3 cells (Fig. 5D) and failed to induce notable mRNA expression of IFN-λ1 (Fig. 5D), IFN-β, or CXCL10 (data not shown). As a control, both live and UV-irradiated influenza B viruses (IBV) induced similar levels of IFN-λ1 expression at 6 h p.i., while UV irradiation destroyed the ability of IAV to induce IFN-λ1 expression (Fig. 5D). The control virus data are consistent with previously published results (39).

### Replication and induction of innate immunity in Calu-3 cells infected by several SARS-CoV-2 strains.

**FIG 6:**
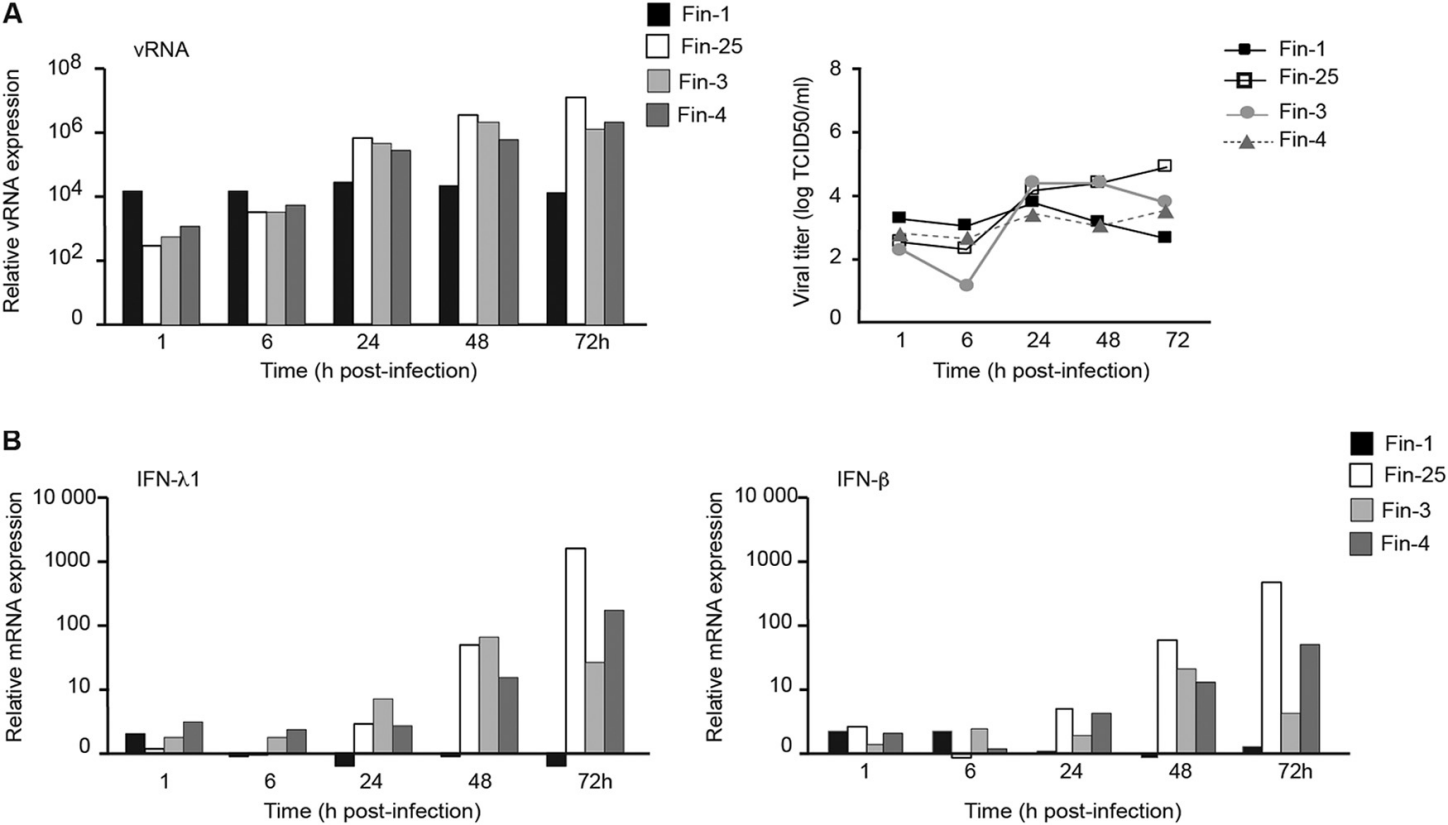
Replication and IFN gene expression in Calu-3 cells infected with four different Finnish SARS-CoV-2 strains. (A) Calu-3 cells were challenged with four different strains of SARS-CoV-2 (Fin-1, Fin-25, Fin-3, and Fin-4) at an MOI of 1, and virus replication was analyzed by qRT-PCR on viral RNA expression in cellular RNA samples and as viral titers in the culture supernatants collected at different time points during a 3-day infection experiment. Viral RNA levels are shown as relative amounts over the mock sample, and viral titers are shown as log TCID_50_/ml. (B) SARS-CoV-2 (Fin-1, Fin-25, Fin-3, and Fin-4)-infected Calu-3 cells were subjected to mRNA measurement of IFN-λ1 and IFN-β at different time points after infection. The results are shown as fold induction over the mock sample.

Next, we compared the ability of four SARS-CoV-2 strains (Fin-1, Fin-25, Fin-3, and Fin-4) to replicate and induce type I and type III IFN responses in Calu-3 cells in order to get a broader view of the characteristics of different SARS-CoV-2 strains. Calu-3 cells were infected with four different strains of SARS-CoV-2 at an MOI of 1, and qRT-PCR was performed on RNA samples collected at different time points after infection. Fin-25, Fin-3, and Fin-4 strains replicated clearly better than the Fin-1 strain in Calu-3 cells, and the replication kinetics of these three strains were very similar (Fig. 6A). The productivity of the infection (TCID_50_ titers) correlated well with viral RNA expression levels (Fig. 6A). The kinetics of IFN-λ1 and IFN-β mRNA expression induced by different viruses were variable, and cytokine gene expression seemed to follow the ability of a given virus to replicate and express viral RNA. Fin-1 virus did not induce IFN-λ1 or IFN-β mRNA expression, while Fin-25, Fin-3, and Fin-4 strains induced clearly detectable mRNA expression of these cytokines (Fig. 6B). Interestingly, the Fin-25 strain appeared to replicate better than the other three viruses, and its ability to induce IFN genes was also the best (Fig. 6B).

## DISCUSSION

The recent COVID-19 pandemic has brought up the fears raised by the SARS epidemic in 2002 to 2003. The high genetic identity of ∼80% between SARS-CoV-2 and SARS-CoV may result in analogous molecular interactions and similar pathogenesis of the two CoVs (17). Unlike in the SARS epidemic, in the COVID-19 pandemic, the novel virus shows differentiation to seven main clades (GISAID, [40]) or hundreds of lineages (phylogenetic assignment of named global outbreak lineages [PANGOLIN], [41]). At the present rate of global spreading, SARS-CoV-2 is accumulating 2 to 3 mutations a month with a maximum of ∼55 amino acid changes to date (GISAID and Nextstrain data), which accounts for less than 0.2% of the genome. However, analysis of the effect of sequence variability on the pathogenesis of different sublineages of SARS-CoV-2 and their ability to regulate host immune responses is of great importance. Presently, there are several studies on SARS-CoV-2 replication in certain stable cell lines (17, 31, 33, 42, 43). However, information on the replication of SARS-CoV-2 in human lung epithelial cells, MΦs, and DCs is still very limited and controversial (44–46), even though these cells are the likely primary target cells of SARS-CoV-2 infection. In the present study, we have demonstrated that the replication of all investigated SARS-CoV-2 sublineages in human monocyte-derived DCs, MΦs, monocytes, and lymphocytes was clearly impaired. This observation resembles the observations of SARS-CoV and MERS-CoV, which do not replicate in these cells (27, 28). However, different sublineages of SARS-CoV-2 possess different replication capacities and abilities to induce innate immune responses in human lung epithelial Calu-3 cells. The SARS-CoV-2 hCoV-19/Finland/FIN-25/2020 strain isolated from a traveler returning from Milan in March 2020 showed the best ability to replicate and induce IFN responses. Moreover, TPCK-treated trypsin treatment enhanced the replication of SARS-CoV-2 in Calu-3 cells but failed to change the abortive nature of the infection in DCs and MΦs.

Consistent with SARS-CoV (47, 48), both Fin-1 and Fin-25 SARS-CoV-2 strains did not seem to be able to infect human monocyte-derived MΦs and DCs, and both strains failed to induce notable cytokine responses in immune cells. Our finding is consistent with other groups showing that infection of SARS-CoV-2 in human immune cells is abortive, and no cytokine response or a weak cytokine response is seen (44, 45). However, another study demonstrated that SARS-CoV-2 could efficiently infect human immune cells, although with an abortive nature (46). Inconsistent with our UV treatment studies, they showed that the incoming viruses, live or heat inactivated, were able to induce notable cytokine gene expression in human immune cells (46). Further studies are warranted to reveal whether these discrepancies are due to different experimental conditions or virus strains used in the analyses. It remains an open question whether weak induction of IFNs by lung epithelial cells and lack of IFN production by immune cells contributes to unrestricted replication of SARS-CoV-2 in the lungs at early stages of infection. As a bridge between innate and adaptive immunity, DCs need to migrate to local lymph nodes to present antigens and activate adaptive immunity. The trafficking of SARS-CoV-2-infected DCs without triggering notable IFN responses could theoretically transfer virus to T cells in lymph nodes or bronchial pneumocytes (49), facilitating virus spread in the host. Currently, there is a general concern of antibody-dependent enhancement (ADE) (50–53) triggered by vaccines or antibody therapies against SARS-CoV-2 infection. Clinical evidence for ADE has not been observed. Our study also showed that SARS-CoV-2 is not replicating in human leukocytes, questioning the possibility of ADE in COVID-19. Lymphopenia observed in COVID-19 patients with severe outcome is likely not due to direct infection of lymphocytes but is rather due to a systemic response to SARS-CoV-2 infection.

SARS-CoV-2 S protein mediates membrane fusion and facilitates the entry of the virus into target cells. The presence of cellular receptors for the attachment of SARS-CoV-2 and priming cleavage of S protein are two key factors for successful entry of the virus (33, 43). Angiotensin-converting enzyme 2 (ACE2) has been demonstrated as the entry receptor for both SARS-CoV (54) and SARS-CoV-2 (33), which uses transmembrane protease serine 2 (TMPRSS2) for S protein priming (33). The expression of ACE2 and other entry receptors of CoVs is seen in cells that are permissive for virus replication. In our study, the abortive infection of SARS-CoV-2 in human monocyte-derived MΦs and DCs may be due to the lack of expression of the ACE2 receptor in these cell types (43, 55). Instead, SARS-CoV-2 replicated in Calu-3 cells in which the ACE2 receptor is expressed (56). Indeed, TPCK-treated trypsin treatment enhanced the cleavage of S protein of both the Fin-1 and Fin-25 strains and thus strengthened the replication of both strains in Calu-3 cells. However, trypsin-treated SARS-CoV-2 failed to replicate in human monocyte-derived MΦs and DCs. Although DC-specific intercellular adhesion molecule 3-grabbing nonintegrin (DC-SIGN) was reported to be an alternative entry receptor of SARS-CoV expressed in DCs and alveolar MΦs (57), it was demonstrated to be less efficient in enhancing the infection of SARS-CoV in immune cells (58). Our study on the lack of SARS-CoV-2 replication in human immune cells is consistent with previous studies that showed that human MΦs and DCs are nonpermissive for SARS-CoV and MERS-CoV replication (27, 28, 47, 48).

Mutations in the S protein gene of SARS-CoV-2 viruses, especially in the receptor-binding domain (RBD) of S protein, are in the highest frequency in the CoV genome (16). Sequence comparison between Fin-1 and Fin-25, which manifested the highest difference in their characteristics in our infection model, showed that the Fin-25 strain of SARS-CoV-2 bears five nucleotide and two amino acid mutations, which are located in RdRp (1 sites) and S (1 site) protein, compared to the reference strain Wuhan-Hu-1 of SARS-CoV-2. In addition, passaging of the SARS-CoV-2 viruses in Vero E6 cells has been shown to trigger fast acquisition of mutations into the furin-like cleavage site of S protein, which was seen in our stock viruses as well (59, 60). Although it has also been demonstrated that trypsin efficiently cleaves the S1/S2 boundary site of S protein of SARS-CoV (61), it is interesting that, despite these mutations, trypsin treatment also greatly enhanced the replication of both SARS-CoV-2 Fin-1 and Fin-25 strains in Calu-3 cells. However, it still remains unclear whether these amino acid changes, and which of them, contribute to the replication ability of SARS-CoV-2 in Calu-3 cells. In the future, it will be important to systematically compare the phenotypic characteristics of SARS-CoV-2 strains during the evolution of the virus.

The ability to replicate and induce IFN and inflammatory responses in Calu-3 lung epithelial cells was different among different isolates of SARS-CoV-2. Stronger and faster activation of IRF3 and p38 phosphorylation and subsequent induction of IFN-λ1 mRNA and MxA protein expression are associated with better replication of Fin-25 virus than Fin-1 virus in Calu-3 cells. Differential virus replication and the ability to induce IFNs and CXCL10 by Fin-1 and Fin-25 as well by Fin-3 and Fin-4 may indicate some adaptation of SARS-CoV-2 to human cells. The ability of SARS-CoV-2 to induce innate immune responses is tightly related to the replication level of the virus, since no immune responses were detected in human immune cells infected with SARS-CoV-2 viruses nor in Calu-3 cells infected with UV-irradiated SARS-CoV-2 viruses.

In summary, our study showed that the phenotypic characteristics of different isolates of SARS-CoV-2 in Calu-3 cells were different, while the replication of all studied SARS-CoV-2 strains in human monocyte-derived MΦs and DCs were abortive, similar to SARS-CoV and MERS-CoV (27, 28). TPCK-treated trypsin treatment to precleave the S protein of SARS-CoV-2 enhanced the replication of SARS-CoV-2 in Calu-3 cells but not in human immune cells. Our study provides new information on the pathogenesis of SARS-CoV-2 in human cells, which can be taken into account in designing the optimal treatment modalities of severe COVID-19 and novel antiviral drugs. The findings will open up the way for further studies on the mechanism of pathogenesis of SARS-CoV-2 in the future.

## MATERIALS AND METHODS

### Cell cultures.

Human primary monocytes were purified from the freshly collected, leukocyte-rich buffy coat layer in centrifuged blood samples obtained from healthy blood donors as described previously (62). PBMCs were obtained after Ficoll gradient centrifugation and were grown in RPMI 1640 medium (Sigma-Aldrich) supplemented with 0.6 μg/ml penicillin, 60 μg/ml streptomycin, 2 mM l-glutamine, 20 mM HEPES, and 10% (vol/vol) fetal bovine serum (Sigma-Aldrich). Lymphocyte and monocyte gradients were obtained from Percoll centrifugation from where monocytes were plated by adhesion and were further differentiated into either MΦs or immature DCs as described previously (63). Nonadherent lymphocytes were used for additional experiments and grown in RPMI 1640 medium with supplements. Monocytes were allowed to adhere to plates (Sarstedt) for 1 h at 37°C in RPMI 1640 medium to obtain monocytes for MΦ differentiation. The cells were washed using cold phosphate-buffered saline (PBS; pH 7.35), and the remaining monocytes were cultured in MΦ/serum-free medium (Life Technologies) supplemented with recombinant human granulocyte-macrophage colony-stimulating factor (GM-CSF) (10 ng/ml; Gibco Invitrogen). Cells were differentiated into MΦs for 6 days, with a change to fresh culture medium every 2 days.

The differentiation of monocyte-derived DCs was achieved by cultivating the adherent monocytes in the presence of 10 ng/ml of recombinant human GM-CSF (Gibco Invitrogen) and 20 ng/ml of recombinant human interleukin-4 (IL-4) (GenScript) in RPMI 1640 medium supplemented as above. The cells were cultivated for 6 days, and fresh medium was added every 2 days.

Cultured human airway epithelial cell lines Calu-3 (ATCC, HTB-55) and Vero E6 green monkey kidney cells (ATCC, CRL-1586) were grown in Eagle minimal essential medium (Eagle-MEM) (Sigma-Aldrich). Cell culture medium was supplemented as described above for RPMI, except for Calu-3 cells where 15% fetal bovine serum was used. All cells were maintained at 37°C in a humidified atmosphere in the presence of 5% CO_2_.

### Viruses and infections.

SARS-CoV-2 virus strains initially named as hCoV-19/Finland/1/2020 (EPI_ISL_407079), hCoV-19/Finland/FIN-25/2020 (EPI_ISL_412971), hCoV-19/Finland/3/2020 (EPI_ISL_2365908), and hCoV-19/Finland/4/2020 (EPI_ISL_2365909) were isolated from the nasopharyngeal samples of COVID-19 patients. SARS-CoV virus strain HKU-39849 (GenBank number AY278491) was provided by the Erasmus Medical Center (Rotterdam, The Netherlands). All the viruses were propagated in Vero E6 cells with a passage history of Vero E6 passage 3 (p3) for Fin-1, Vero E6 p3 for Fin-25, Vero E6 p2 for Fin-3 and Fin-4, and Vero E6 pXp2 for SARS-CoV to obtain virus stocks for the experiments. The titers of SARS-CoV-2 and SARS-CoV stocks were determined to be 1.5 × 10^6^, 1.5 × 10^7^, 1 × 10^7^, 1 × 10^8^, and 2.5 × 10^7^ TCID_50_/ml, respectively, as determined by an endpoint dilution assay in Vero E6 cells.

The primary human immune cells and Calu-3 and Vero E6 cell lines were infected with the indicated SARS-CoV-2 or SARS-CoV strains at different MOI values (based on TCID_50_ Vero E6 cell titers), as shown in the figures. Infective SARS-CoV-2 and SARS-CoV viruses were handled under biosafety level (BSL) 3 laboratory conditions at the Finnish Institute for Health and Welfare (THL), Finland.

Human influenza A virus A/Beijing/353/89 (H3N2) and influenza B virus B/Shangdong/7/97 were grown for 3 days at 36°C in allantoic cavities of 11-day-old embryonated chicken eggs. Cells were infected with influenza viruses at an MOI of 1 for different time points as indicated in the figures. UV irradiation of the viruses was performed by 600 mJ of UV light before adding the viruses on the cells.

### Endpoint dilution assay.

For determining the viral titers in SARS-CoV-2 and SARS-CoV samples, Vero E6 cells were cultured in 96-well plates. A dilution series was made from each sample, and each dilution was used to infect eight parallel culture wells. The cytopathic effect was observed under a light microscope at day 3 p.i., and each well was scored either positive or negative for virus infection. The Spearman-Kärber method was used to calculate the results, which are presented as log TCID_50_/ml.

### Antibodies against SARS-CoV N protein and SARS-CoV-2 S1 protein.

SARS-CoV N protein- and SARS-CoV-2 S1 protein-specific rabbit antibodies were prepared against baculovirus-expressed preparative SDS-PAGE-purified SARS-CoV N protein (27) or HEK293 cell-produced S1 (64). Three New Zealand White rabbits were immunized four times with 50 μg/dose/rabbit of N or S1 protein at 4- or 3-week intervals, respectively. The animals were bled 10 days after the last immunization.

### Immunofluorescence assay.

For immunofluorescence microscopy, cells were grown on glass coverslips for 24 h and infected with SARS-CoV or SARS-CoV-2 as indicated in the figure legend. After infection, the cells were fixed with 4% paraformaldehyde at room temperature for 30 min and permeabilized with 0.1% Triton X-100 for 5 min before staining with anti-SARS-CoV N protein-specific antisera. Polyclonal rabbit immune serum was used at a dilution of 1:200 for staining the coverslips at room temperature for 1 h. Fluorescein isothiocyanate (FITC)-labeled goat anti-rabbit antibodies (Invitrogen) were used in secondary staining, and slides were analyzed with a Leica TCS NT confocal laser microscope.

### RNA isolation and qRT-PCR.

Virus-infected cells were harvested, and total cellular RNA was isolated using the RNeasy mini kit (Qiagen) including DNase digestion (RNase-free DNase kit, Qiagen). Total cellular RNA (500 ng) was transcribed to cDNA using a TaqMan reverse transcriptase kit (Applied Biosystems) with random hexamers as primers. cDNAs were amplified by PCR using TaqMan universal PCR master mix and gene expression assays (Applied Biosystems).

The SARS-CoV-2 E gene (65), IAV M1 gene, or IBV NP gene-specific qRT-PCR primers (39) were used for analyzing viral RNA expression, and commercially available primers (Applied Biosystems) were used for analyzing the expression levels of IFN-α, IFN-β, IFN-λ1, CXCL10, IL-1β, IL-6, IL-8, and ΤΝF-α mRNAs. Cytokine mRNA levels were normalized against human 18S rRNA with TaqMan endogenous control kits (Applied Biosystems). Gene expression data are presented as relative gene expression in relation to unstimulated samples in order to calculate the fold changes seen in infection experiments.

### Sequencing.

Viral RNA extracted from original swab samples and virus culture using the RNeasy minikit (Qiagen) was reverse transcribed to cDNA using a LunaScript RT SuperMix kit (New England Biolabs). Primer pools targeting SARS-CoV-2 were designed using the PrimalScheme tool (66), and PCR was conducted using PhusionFlash PCR master mix (Themo Fisher). Sequencing libraries were prepared using an NEBNext Ultra II FS DNA library kit (New England Biolabs) according to the manufacturer’s instructions and were sequenced using an Illumina Miseq with a v3 sequencing kit. Raw sequence reads were trimmed, and low quality (quality score of <30) and short (<25 nt) sequences were removed using Trimmomatic (67). The trimmed sequence reads were assembled to the reference sequence (NC_045512.2) using the BWA-MEM (68) algorithm implemented in SAMTools version 1.8 (69). Mutation frequencies in virus populations were estimated based on minority variant calling with LoFreq (70).

### Phylogenetic analysis.

To analyze the sequences of the Finnish isolates, a data set comprising the reference sequence (NC_045512.2) and six sequences representing the major early clades (A, B, B.1, B.1.1, and B.2) were retrieved from GISAID (https://www.gisaid.org). Accession codes and sources for all of the sequences used in the phylogenetic analysis of the four Finnish SARS-CoV-2 isolates are described in the Data availability section. Sequences were aligned using multiple sequence comparison by log expectation (MUSCLE [71]) software implemented in the molecular evolutionary genetics analysis computing platform (MEGA 7 [72]). Based on the estimation for the best model for the data set in MEGA 7, the phylogenetic tree was constructed by the maximum likelihood method (73). Support for the phylogenies was estimated with 1,000 bootstrap replicates.

### Immunoblotting.

For protein expression analyses, cells from different blood donors were pooled to obtain sufficient amounts of protein. The whole-cell lysates from cell lines or pooled primary cells were prepared in the passive lysis buffer of the dual luciferase assay kit (Promega) containing 10 mM Na_3_PO_4_. Equal amounts of protein (10 to 30 μg/lane) were separated by SDS-PAGE and transferred to Hybond-P polyvinylidene difluoride (PVDF) membranes (Amersham Biosciences). The membranes were blocked with 5% milk protein in PBS. Antibodies against IRF3 and MxA were as previously described (74, 75), and antibodies against SARS-CoV N protein and SARS-CoV-2 S1 protein were prepared as described above. Staining was done in blocking buffer at room temperature for 1 h. Antibodies against phosphorylated IRF3 (P-IRF3; 4947), p38 (9212), phosphorylated p38 (P-p38; 9211L), and glyceraldehyde-3-phosphate dehydrogenase (GAPDH; 2118) were from Cell Signaling Technology, and staining was done in Tris-buffered saline, pH 7.4, containing 5% bovine serum albumin (BSA) at 4°C overnight. Horseradish peroxidase (HRP)-conjugated antibodies (Dako) were used in the secondary staining at room temperature for 1 h. Protein bands were visualized on HyperMax films using an ECL plus system (GE Healthcare).

### ELISA.

IFN-λ1 levels from cell culture supernatants were determined using a LegendMax human IFN-λ1 ELISA kit (BioLegend) according to the manufacturer’s instructions.

### Flow cytometry.

For determining the infectivity of SARS-CoV-2 viruses in the subpopulations of human PBMCs, cells from four different blood donors were harvested and handled separately. Cells were fixed with 4% paraformaldehyde (PFA) for 30 min, permeabilized with 0.1% Triton X-100 for 5 min, and treated with 0.5% BSA in PBS. Virus-infected cells were double stained with antibodies for specific cell surface markers CD14-peridinin chlorophyll protein (PerCP)/Cy5.5, CD3-FITC, or CD19-FITC (BioLegend) and viral antigens (rabbit anti-SARS-CoV N or rabbit anti-IAV [74]). The secondary antibodies were phycoerythrin (PE)-conjugated anti-rabbit antibody (Thermo Fisher Scientific). In all antibody stainings, the cells were stained at room temperature for 1 h and washed twice with 0.5% BSA in PBS. The samples were analyzed with a FACSCanto II (BD) device using FACSDiva software.

### Quantification and statistical analyses.

Unless otherwise stated, experiments were performed at least three times with cell lines or with primary cells from four individual blood donors, and data are presented as means ± the standard error of the means (SEM).

### Ethics statement.

Adult human blood-derived leukocytes used in the experiments were obtained from anonymous healthy blood donors through the Finnish Red Cross Blood Service. The use of buffy coats for research purposes was approved by the Finnish Red Cross Blood Service institutional review board (license number 35/2021, renewed yearly) by which the need for informed consent was waived. Ethical approval for animal immunization was provided by the ethics committee of animal experimentation in Southern Finland (permission number ESLHESAVI/11411/04.10.07/2014 to Anna Meller). All experimental protocols were approved and performed in accordance with the guidelines of the Finnish Institute for Health and Welfare (THL), Helsinki, Finland.

### Data availability.

The following are the originating laboratories that were responsible for obtaining the specimens and the submitting laboratories where genetic sequence data were generated and shared via the GISAID Initiative: hCoV-19/Wuhan/WH04/2020, EPI_ISL_406801, General Hospital of Central Theater Command of People's Liberation Army of China, BGI, Institute of Microbiology, Chinese Academy of Sciences, Shandong First Medical University, Shandong Academy of Medical Sciences, and General Hospital of Central Theater Command of People’s Liberation Army of China; hCoV-19/Australia/VIC138/2020, EPI_ISL_419834, Victorian Infectious Diseases Reference Laboratory (VIDRL) and Victorian Infectious Diseases Reference Laboratory and Microbiological Diagnostic Unit Public Health Laboratory, Doherty Institute; hCoV-19/Hangzhou/HZCDC0167/2020, EPI_ISL_421223, Hangzhou Center for Diseases Control and Prevention; hCoV-19/England/200940527/2020, EPI_ISL_414005, Respiratory Virus Unit, Microbiology Services Colindale, Public Health England; hCoV-19/France/HF1645/2020, EPI_ISL_418220, Centre Hospitalier Compiègne Laboratoire de Biologie and National Reference Center for Viruses of Respiratory Infections, Institut Pasteur, Paris; hCoV-19/Zhejiang/HZ103/2020, EPI_ISL_422425, Zhejiang Provincial Centers for Disease Control and Prevention and Zhejiang Provincial Centers for Disease Control and Prevention; NC_045512.2 Shanghai Public Health Clinical Center and School of Public Health, Fudan University, Shanghai, China.
